# The Role of Food Addiction and Lifetime Substance Use on Eating Disorder Treatment Outcomes

**DOI:** 10.3390/nu15132919

**Published:** 2023-06-27

**Authors:** Romina Miranda-Olivos, Zaida Agüera, Roser Granero, Susana Jiménez-Murcia, Montserrat Puig-Llobet, Maria Teresa Lluch-Canut, Ashley N. Gearhardt, Fernando Fernández-Aranda

**Affiliations:** 1 CIBER Fisiopatología Obesidad y Nutrición (CIBERobn), Instituto de Salud Carlos III, 28029 Madrid, Spain; rmiranda@idibell.cat (R.M.-O.);; 2 Clinical Psychology Unit, L’Hospitalet de Llobregat, Hospital Universitari de Bellvitge, 08907 Barcelona, Spain; 3 Psychoneurobiology of Eating and Addictive Behaviors Group, Neurosciences Programme, Bellvitge Biomedical Research Institute (IDIBELL), L’Hospitalet de Llobregat, 08908 Barcelona, Spain; 4 Departament d’Infermeria de Salut Pública, Salut Mental i Materno-Infantil, Escola d’Infermeria, Facultat de Medicina i Ciències de la Salut, L’Hospitalet de Llobregat, Universitat de Barcelona (UB), 08007 Barcelona, Spain; monpuigllob@ub.edu (M.P.-L.); tlluch@ub.edu (M.T.L.-C.); 5 Research Group in Mental Health, Psychosocial and Complex Nursing Care (NURSEARCH), Facultat de Medicina i Ciències de la Salut, L’Hospitalet de Llobregat, Universitat de Barcelona (UB), 08007 Barcelona, Spain; 6 Departament de Psicobiologia i Metodologia de les Ciències de la Salut, Universitat Autònoma de Barcelona, 08193 Barcelona, Spain; 7 Department of Clinical Sciences, School of Medicine and Health Sciences, University of Barcelona, 08007 Barcelona, Spain; 8 Department of Psychology, University of Michigan, Ann Arbor, MI 48109, USA; agearhar@umich.edu

**Keywords:** food addiction, substance use, treatment outcomes, eating disorders

## Abstract

Food addiction (FA) and substance use (SU) in eating disorders (ED) have been associated with a more dysfunctional clinical and psychopathological profile. However, their impact on treatment outcomes has been poorly explored. Therefore, this transdiagnostic study is aimed at examining whether the presence of FA and/or SU is associated with treatment outcomes in patients with different ED types. The results were not able to reveal significant differences in treatment outcomes between patients with and without FA and/or SU; however, the effect sizes suggest higher dropout rates in the group with both FA and SU. The predictive models of treatment outcomes showed different features associated with each group. High persistence (i.e., tendency to perseverance and inflexibility) was the personality trait most associated with poor treatment outcomes in patients without addictions. High harm avoidance and younger age at ED onset were the variables most related to poor outcomes in patients with FA or SU. Finally, in the group with both addictive behaviors (FA and SU), the younger patients presented the poorest outcomes. In conclusion, our results suggest that, regardless of presenting addictive behaviors, patients with ED may similarly benefit from treatment. However, it may be important to consider the differential predictors of each group that might guide certain treatment targets.

## 1. Introduction

The comorbidity between eating disorders (ED) and some addictive-related patterns, such as food addiction (FA), behavioral addictions, and substance use (SU), has been widely described in the literature. The construct of FA combines the concepts of substance-based and behavioral addictions. Thus, there is no consensus on whether FA should be integrated within substance use disorders (SUDs) [1] or, on the contrary, within behavioral addictions [2]. However, many studies increasingly point out that the mere application of criteria based on the addictive chemical properties of food (as in SU) does not seem sufficient to fully capture the phenomenological aspects of an FA, hence the need to study FA and SU as distinct addictive behaviors [3]. A high prevalence rate of FA has been widely reported in individuals with obesity and/or ED, mainly in those with bulimia nervosa (BN) and binge eating disorder (BED) (70–90%) [4,5]. Intriguingly, the presence of FA has also been described in patients with anorexia nervosa (AN), especially in those of the bulimic-purging subtype (AN-BP), compared to the restricting subtype (AN-R) (75.0% and 54.2%, respectively) [6,7,8]. The presence of FA in patients with ED has been associated with greater severity of ED-related symptomatology (e.g., a higher frequency of binge-eating episodes, emotional eating, and compulsive eating) [5,7,8,9], as well as more dysfunctional personality traits (i.e., high impulsivity), and greater general psychopathology (mainly more depressive symptoms) [5,8]. The impact of FA on the prognosis of EDs has not yet been fully investigated. Hilker et al. [10] reported that the severity of both FA and binge/purge episodes decreased after a psychoeducational program in patients with BN. Likewise, Romero et al. [4] found that the association between FA and treatment outcomes appeared to be mediated by the severity of the ED. Additionally, this study found that FA was indirectly associated with poorer treatment outcomes in BED, but not in BN [4]. There are currently no studies exploring the impact of FA on the prognosis of AN. However, a recent study suggested that FA in AN-R could increase the likelihood of a crossover diagnosis to AN-BP [6].

In terms of SU in ED, numerous studies over the last years have addressed this topic of interest, reporting SU prevalence rates in EDs ranging from 21% to 50%, with tobacco being the most prevalent addictive substance, but also caffeine, alcohol, and illicit drugs [11]. The comorbidity of lifetime SU varies among the ED diagnoses, being more prevalent in BN (34%), followed by BED (18%), AN (13%), and other specified feeding or eating disorders (OSFED) (12%) [8] The coexistence of EDs and SU has been associated with high levels of impulsivity and sensation-seeking that may promote addictive-like behaviors towards a stimulus perceived as rewarding [12,13]. Additionally, SU in BN has been linked to increased emotional dysregulation, suggesting that certain substances might be used as a maladaptive strategy to cope with negative emotions [14,15] The purpose of substance misuse in patients with ED is a relevant aspect to consider [16]. It is important to identify whether SU is part of the ED symptomatology as a strategy aimed at weight control (e.g., caffeine, tobacco, psychostimulant substances), or whether it acts as a maladaptive coping mechanism to deal with negative emotions (e.g., alcohol, psychoactive substances) [16,17]. In both cases, the functional role of SU may guide different targeted intervention strategies [11]. In addition, the co-occurrence of ED with SU has been associated with a poorer prognosis, contributing to a longer duration of the disorder, greater psychopathology and presence of other psychiatric disorders, and an increased risk of mortality [18,19]. This co-occurrence may accentuate the symptomatology of ED, hampering its recovery [11]. However, research directly testing the role of SU on ED treatment outcomes is limited.

The comorbidity of FA and SU in ED have usually been addressed separately. Nevertheless, some studies have indicated the presence of a potential common brain substrate in FA and SU, suggesting shared neurobiological vulnerabilities and genetic predispositions [20]. For instance, certain polymorphisms of the dopamine (DA) receptor D2 have been linked to an increased risk for both FA and SU [21,22]. Considering that the DA system plays a role in appetite regulation and reward pathways, it is plausible that this neurotransmitter system underlies maladaptive eating behaviors in EDs. Based on this premise, it is plausible to hypothesize that the concurrent occurrence of multiple addictive behaviors could have epidemiological implications on treatment outcomes in EDs. However, to date, there have been no studies that have explored the potential effects that the simultaneous presence of both addictive-related patterns could have on treatment outcomes in individuals with EDs. One recent study by our research group explored the joint involvement of FA and/or lifetime problematic alcohol and illicit drug use in EDs [23]. Patients with at least one addictive-like behavior (either current FA or lifetime SU) exhibited more general psychopathology and ED-related symptomatology than those without FA and/or SU. However, the cross-sectional design of this study was not able to address whether this dysfunctional clinical and personality profile has implications for treatment outcomes. Therefore, the main goals of this study were threefold: (a) to examine the sociodemographic and clinical characteristics of patients with different types of ED (including AN, BN, BED, and OSFED) who reported FA and/or lifetime problematic alcohol and illicit drug use (i.e., between those with both addictive behaviors (FA+ and SU+), those with only one addictive behavior (FA+ or SU+), and those with no addictive behaviors (FA− and SU−)); (b) to analyze whether there are differences in treatment outcomes, including dropout rates, between patients with and without current FA and/or lifetime problematic SU; and (c) to describe predictors of treatment outcomes in patients with or without addictive-like behaviors.

## 2. Materials and Methods

### 2.1. Participants

A total of 303 patients with ED composed the sample of this study (32 AN, 132 BN, 67 BED, and 72 OSFED). All patients were attended to at the ED Unit of the Hospital Universitario de Bellvitge (Barcelona, Spain) for assessment and treatment.

### 2.2. Instruments

In the assessment process, sociodemographic and clinical data were collected through a semi-structured face-to-face interview using the SCID-5 [24]. Clinical data covered lifetime alcohol or illegal drug misuse by utilizing module E of the SCID-5 [24]. For the present study, problematic lifetime SU was defined as a problematic pattern of continued use of alcohol and/or illicit drugs at some time throughout the patient’s life, and with negative consequences. These consequences may comprise, for example, higher consumption than intended; craving to, or unsuccessful efforts to control, consumption; consumption despite physical or psychological problems; interpersonal problems; being in dangerous situations; etc. Furthermore, the usually applied questionnaires in the field of EDs were administered, namely:

- The Yale Food Addiction Scale 2.0 (YFAS 2.0), Spanish validation [25]. This is a 35-item, self-report questionnaire to assess FA, based on 11 substance-dependence-related symptoms and adapted to the context of food consumption. This scale allows the classifying of FA into binary categories, namely present (at least 2 symptoms and self-reported clinically significant impairment or distress) and absent. The internal consistency of our sample was excellent (α = 0.97).
- The Eating Disorder Inventory-2 (EDI-2), Spanish validation [26]. It is a 91-item, self-reported questionnaire that assesses 11 ED-related cognitive and behavioral domains. A total score is also provided to report overall ED severity. For this sample, the internal consistency was excellent (α = 0.94).
- The Symptom Checklist-90 Revised (SCL-90-R), Spanish validation [27]. The questionnaire is composed of 90 items that assess 9 dimensions of psychopathology: somatization, obsession–compulsion, interpersonal sensitivity, depression, anxiety, hostility, phobic anxiety, paranoid ideation, and psychoticism. Additionally, it includes three global indices related to overall psychological distress (i.e., the global severity index, GSI), the intensity of symptoms (i.e., the positive symptom distress index, PSDI), and self-reported symptoms (i.e., a total of positive symptoms). The questionnaire demonstrated excellent internal consistency, with a Cronbach’s alpha coefficient of 0.97.
- The Temperament and Character Inventory Revised (TCI-R), Spanish validation [28]. It is a self-reported assessment, consisting of 240 items, that evaluates 4 temperament dimensions (harm avoidance, novelty seeking, reward dependence, and persistence) and 3 character dimensions (self-directedness, cooperativeness, and self-transcendence). Our internal consistency ranged from α = 0.81 to α = 0.89.
- The Impulsive Behavior Scale (UPPS-P), Spanish validation [29]. This self-report questionnaire consists of 59 items that assess 5 distinct facets of impulsivity: positive and negative urgency (a tendency to act rashly in response to positive mood or distress), lack of perseverance (an inability to sustain focus on a task), lack of premeditation (acting without considering the consequences of an action), and sensation seeking (a tendency to seek novel and exciting experiences). The internal consistency for the sample ranged from very good (negative urgency α = 0.83) to excellent (positive urgency α = 0.92).

### 2.3. Treatment

Treatment for BN, BED, and OSFED was provided by experienced psychologists through 16 weekly outpatient group therapy sessions of 90 min each. Separate therapeutic groups were conducted for patients with different diagnoses, but all were on the same cognitive–behavioral therapy (CBT) program. Patients diagnosed with AN (not requiring inpatient treatment for underweight) underwent a day hospital treatment program, which included ten weekly CBT group sessions for about 3 months.

Upon discharge, patients were assessed and assigned into one of three previously defined categories: full remission, partial remission, and non-remission. Criteria for full remission were based on the DSM-5, indicating a complete absence of ED symptoms for at least four weeks of treatment. Partial remission referred to substantial symptomatic improvement with residual symptoms, while non-remission was used to describe poor outcomes. These categories have been employed to evaluate the efficacy of ED treatments in previous studies [4,30,31,32]. Treatment discontinuation was classified as dropout, defined as being absent for at least three consecutive therapy sessions. In addition, patients were also subsequently recategorized into a dichotomous variable (i.e., good versus poor outcomes), which has been previously used to examine ED treatments [33], in order to facilitate the interpretation of the results, especially in the predictive models, namely good outcomes (i.e., full and partial remission) and poor outcomes (i.e., non-remission and dropout).

### 2.4. Statistical Analyses

Stata 17 for Windows was used for the statistical analysis. The comparison between the groups was performed using chi-square tests (χ^2^) for categorical variables, and analysis of variance (ANOVA) for quantitative variables. Bonferroni’s correction was applied for post hoc comparisons in chi-square tests, while post hoc pairwise comparisons for ANOVA were performed using the Bonferroni method. Effect size was considered in the range of mild-moderate to large-high when |CV| > 0.15, mild-moderate if OR > 1.86, and large-high when OR > 3.00. The comparisons of the treatment outcomes (0 = good versus 1 = poor) between the groups were performed by odds ratio coefficients (OR), obtained in logistic regression, and adjusted by the ED type. Stepwise logistic regressions were also used to identify the significant predictors of the treatment outcomes (0 = good versus 1 = poor) for the list of measures at the baseline. Given the exploratory approach of this analysis, we tried to include the maximum number of predictors, while minimizing adjustment problems due to high collinearity. For this reason, in addition to the sociodemographic profile and the personality profile, the global measures associated with the severity of the ED (EDI-2 total), the psychopathological state (SCL-90R GSI), and impulsivity (UPPS-P total) were included. The goodness-of-fit was assessed with the Hosmer–Lemeshow test, and the global predictive capacity with the pseudo-Nagelkerke R^2^ coefficient.

## 3. Results

### 3.1. Descriptive of the Sample

Most participants in the study were women (*n* = 277, 91.4%), single (*n* = 201, 66.3%), or married (*n* = 65, 21.5%), with primary (*n* = 114, 37.6%) or secondary (*n* = 140, 46.2%) education levels, and employed (*n* = 170, 56.1%). The mean age was 32.3 years (SD = 12.3), the mean age of onset of the ED was 20.7 years (SD = 9.9), and the mean duration of the ED was 12.1 years (SD = 9.6).

Table S1 (see Supplementary Materials) includes the comparison of the sociodemographic data, the onset of the ED, the duration, and the BMI between the groups defined by the ED types. Table S1 also contains the distribution of FA, SU, and both FA + SU for each diagnostic type.

Table 1 shows the comparisons between the groups defined by the presence of FA and/or SU. These analyses were performed with bivariate analyses, separately assessing the associations of each variable (defined in the rows) with the created groups (displayed in the columns). Results of these analyses contribute to the first objective of the study. No differences between the groups were found for gender, civil status, education, onset of the ED, and BMI. Differences between the groups were found for the employment status, age, and duration of the ED. The distribution of the ED subtypes was also different for the FA and SU groups being the OSFED condition most associated with FA− and SU−, and BN in the presence of FA+ and/or SU+.

### 3.2. Association of the Treatment Outcomes with the Presence of FA and SU

Table 2 shows the distribution of the treatment outcomes classified into four categories, namely dropout, non-remission, partial remission, and full remission for each group, defined by the presence of FA and/or lifetime SU. Results of these analyses contribute to the second objective of the study. The results of the chi-square test comparing the treatment outcomes between the clinical conditions showed no statistical differences, but an effect size in the moderate range was found comparing the treatment outcomes between FA+ and SU+, and FA− and SU−. The comorbid condition of FA+ and SU+ was associated with a higher risk of dropout. The other comparisons in Table 2 obtained non-significant results and poor effect sizes.

Table 3 displays the distribution of treatment outcomes categorized into two levels, namely poor outcome and good outcome. A logistic regression model was employed, with the group serving as the independent variable, the treatment outcome as the criterion (i.e., dependent variable), and the ED type as a covariate. These analyses contribute to addressing the second objective of the study. Adjusted for the ED type, no statistically significant differences were observed in the pairwise comparisons (effect size was also in the low range).

### 3.3. Predictors of the Treatment Outcomes in the Study

Table 4 presents the results of the stepwise logistic regression analyses conducted to identify the significant predictors of treatment outcomes for each group. The list of potential predictors (independent variables) were the ED types, sociodemographic data (i.e., gender, civil status, education, employment status, and age), onset, duration of the ED, and clinical profile considering the ED symptomatology (EDI-2 total), general psychopathology (SCL-90R), impulsivity levels (UPPS-P total), and personality traits (TCI-R scales). The criterion in the model (dependent variable) was represented by 1 = poor outcome (dropout and non-remission) and 0 = good outcome (partial and full remission). Then, three separate models were tested for each group: the FA− and SU− group (*n* = 56), the FA+ or SU+ group (*n* = 159), and the FA+ and SU+ group (*n* = 88). The results of these analyses contributed to addressing the third objective of the study.

Significant predictors that referred to a higher likelihood for a poor outcome in each group were: (a) high persistence levels (TCI-R) in the FA− and SU− group; (b) an earlier onset of the ED and higher harm avoidance level (TCI-R) in the FA+ or SU+ group; and (c) being younger in the FA+ and SU+ group. These three logistic models in Table 4 achieved an adequate fit (*p* > 0.05 in the Hosmer–Lemeshow tests) and global predictive capacity, considering a Nagelkerke R^2^ coefficient of between 0.08 and 0.17.

Table S2 (see Supplementary Materials) includes comparisons of good and poor outcomes in groups defined for the presence or absence of current FA and lifetime SU. For the FA− and SU− group, a poor treatment outcome was associated with lower levels of body dissatisfaction (EDI-2), lack of perseverance (UPPS-P), harm avoidance (TCI-R), and higher levels of persistence (TCI-R). For the FA+ or SU+ group, a poor treatment outcome was related to higher scores in the EDI-2 ineffectiveness, TCI-R impulse regulation, a poor psychopathological state in the SCL-90-R (interpersonal sensitivity, depression, psychotic, GSI, and PSDI), and a higher TCI-R harm avoidance. For the group with FA+ and SU+, no differences between patients with good and poor treatment outcomes were found.

Table S3 (see Supplementary Material) presents the results for the subsample of patients who had a poor treatment outcome (*n* = 149). The pairwise comparisons between the FA+ or SU+ group versus the FA+ and SU+ group revealed few statistical differences. Notably, statistical differences were observed in the UPPS-P impulsivity measures and TCI-R novelty seeking, with higher scores in the FA+ and SU+ group compared to the FA+ or SU+ group. In contrast, the FA− and SU− group, when compared to the other conditions, exhibited lower scores in the EDI-2, SCL-90-R, and UPPS-P scales. Additionally, their personality profile was characterized by lower novelty seeking and harm avoidance, as well as higher persistence and self-directedness.

Table S4 (see Supplementary Materials) includes a comparison of the treatment outcomes of the three groups stratified by the ED type. No statistical differences were found, but a moderate effect size was found in the AN group, suggesting that the comorbid condition of FA+ and SU+ in AN was associated with an increased risk of a poor treatment outcome.

## 4. Discussion

In recent years, there has been increasing interest in understanding the role of FA and other comorbid addictions on the clinical status and prognosis of patients with ED. However, to the best of our knowledge, this is the first study that aims to address whether the presence of one (i.e., FA or SU) or multiple lifetime addictive behaviors (i.e., FA and SU) might have an impact on the treatment outcomes of these patients. Because most patients with an ED have a co-occurrence with some addictive behavior, it is essential to study them to understand and consider how the addiction perspective can be included in the care of these patients. Intriguingly, the results suggest that there are no differences related to the presence of addictive-like behaviors, in terms of treatment outcomes of patients with ED. However, despite not reaching statistical significance, a moderate effect size was observed in the group with both FA and SU, in terms of higher dropout rates than the non-addiction group.

Similarly to previous findings [23], when comparing patients with a poor treatment outcome, we found that those with at least one addiction-related behavior were characterized by greater overall psychopathology, more severity of ED, and more dysfunctional personality traits, compared to the non-addiction group. However, in contrast with our hypothesis, our findings did not show significant differences in treatment outcomes between individuals without addictive behaviors and those with FA and/or SU. These results are in line with a previous study reporting that FA mediates the severity of ED but is not, per se, directly associated with the treatment response [4]. However, despite the absence of significant results, when considering diagnostic types separately, the effect size of the comparisons suggests that the FA+ and SU+ condition was associated with an increased risk of poor treatment outcomes in patients with AN. Further studies with higher sampling power are needed to corroborate this hypothesis.

We also hypothesized that patients with one or more addictive behaviors would have higher dropout rates; however, no statistically significant differences were found between the three groups. This finding is in line with another study that found no differences in treatment outcomes between patients with ED who were either with or without SU disorder [16]. However, it is not consistent with other studies that found greater treatment dropout rates in patients with addictions [34,35]. In this vein, it should be noted that these previous studies were conducted with current SU disorders, and not with lifetime problematic SU, as in this current study, which could explain the lack of differences. Nonetheless, we observed a moderate effect size, suggesting the comorbid condition of FA+ and SU+ was associated with a higher risk of dropout. It might represent a very relevant point for clinical practice that deserves further investigation. For instance, it would be interesting to identify in which treatment session they are most likely to drop out in order to identify strategies to reduce dropout, as well as to include relevant treatment goals from the early stages of care, and to discuss addiction.

Regarding predictive models of treatment outcome, our results showed different features associated with a poor outcome in each group. Firstly, for the group with no addictive-related behavior (i.e., FA− and SU−), a higher persistence (i.e., higher perfectionism and tendency to perseverance and inflexibility) was associated with a poor outcome. Although some studies have reported that a higher persistence is related to more adherence to treatment and less risk of dropout [36], other studies have related a high persistence to the risk of non-remission of the symptomatology, and even relapse, due to the perpetuation of ED-related maladaptive behaviors [37,38,39,40,41]. In this regard, persistence might be linked to the motivational processes of these patients. That is, if they are motivated to initiate treatment, persistence could act as a positive factor related to treatment adherence, despite the considerable effort involved. However, if patients are maladaptively motivated to perpetuate ED-related behaviors, persistence may be associated with a poorer treatment outcome.

Secondly, higher harm avoidance, and an earlier onset of the disorder, were predictors of poor treatment outcomes in patients who reported FA+ or SU+. Overall, a higher harm avoidance has been previously associated with a positive FA score [25], and with other comorbid addictions [42,43,44]. Some authors have proposed that the link between harm avoidance and outcomes is based on a lack of functional coping strategies, increasing the risk of engaging in dysfunctional patterns in food or drug consumption [45,46]. Therefore, the decrease of harm avoidance through interventions, based on the promotion of adaptive coping strategies in response to distressing events, could be a good therapeutic target to reduce the risk of engaging in addictive patterns as well. The relationship between the age of the onset of ED and a poor treatment outcome is consistent with previous studies postulating that a younger age of onset of ED may increase its severity and even the risk of presenting comorbidities such as SU [47,48]. In addition, it has also been associated with greater difficulties in adapting to the treatment recommendations [49]. Finally, poor treatment outcomes in patients with both addictive-related behaviors were associated with younger age at the time of treatment. This may be related to the postulation that younger people are more prone to impulsivity [50] and, therefore, could be more prone to engage in addictive behaviors [51,52].

### Limitations and Strengths

The current study has some limitations that should be considered. First, one has to be cautious in interpreting these results related to lifetime SU, as our results refer to patients with a history of substance misuse having a non-full diagnosis of SU disorder. Second, all participants were recruited from a hospital setting and, therefore, the results may not be representative of the entire population with EDs. Finally, the sample size did not allow for meaningful comparisons between groups to assess the severity of SU, or compare between alcohol and other illicit drug users. In addition, it would be interesting to consider how many different substances the participants had consumed in their lifetime, data that were not available in this study. Further research with larger sample sizes should analyze these shortcomings, as well as analyze whether there are differences between the FA+ and SU- and the FA- and SU+ groups, which were not possible to analyze in this study, due to the low proportion of patients with ED and lifetime SU but without FA (*n* = 6). Despite these limitations, the current study also presents several strengths that should be considered. In terms of treatment outcomes, identifying predictors of each group (FA+ and SU+, FA+ or SU+, and FA− and SU−) would improve our ability to better understand the differences related to these patients’ profiles and, thus, provide better treatment options. Further research is needed to investigate this further.

## 5. Conclusions

In conclusion, it is noteworthy that most patients with EDs suffered from FA and/or lifetime SU, and only the minority did not represent an addictive-related phenotype. Our results did not show significant differences between the three groups in terms of treatment outcomes. However, the effect sizes, when comparing the FA+ and SU+ group with those who did not present any addictive behaviors, suggest higher dropout rates in the former. Additionally, when comparing those patients with poor outcomes, we found that those with at least one addiction-related behavior also present greater symptomatological and psychopathological severity, as well as more dysfunctional personality traits, ratifying the clinical importance of screening for the presence of addictive behavioral patterns in EDs. Furthermore, distinctive predictors were found in each group, highlighting the need to select specific therapeutic strategies to improve the efficacy and adherence to treatments in these patients with different addictive profiles. Further studies are also needed to analyze the possible genetic predisposition to FA and SU in these patients, with special attention to dopamine receptor genes.

## Figures and Tables

**Table 1:** Descriptive statistics of the sample based on the presence of FA and SU.

		FA− and SU− *n* = 56		FA+ or SU+ *n* = 159		FA+ and SU+ *n* = 88			
		*n*	%	*n*	%	*n*	%	*p*	Groups with Significant Differences
Gender	Women	49	87.5%	148	93.1%	80	90.9%	0.430	
	Men	7	12.5%	11	6.9%	8	9.1%		
Civil status	Single	42	75.0%	99	62.3%	60	68.2%	0.413	
	Married/partner	9	16.1%	40	25.2%	16	18.2%		
	Divorced/separated	5	8.9%	20	12.6%	12	13.6%		
Education	Primary	23	41.1%	55	34.6%	36	40.9%	0.539	
	Secondary	26	46.4%	73	45.9%	41	46.6%		
	University	7	12.5%	31	19.5%	11	12.5%		
Employment	Unemployed	13	23.2%	30	18.9%	17	19.3%	0.001 *	All pairwise comparisons with *p <* 0.05
	Student	23	41.1%	41	25.8%	9	10.2%		
	Employed	20	35.7%	88	55.3%	62	70.5%		
		Mean	SD	Mean	SD	Mean	SD	*p*	
Age (years old)		29.04	12.90	32.56	12.56	34.19	10.93	0.046 *	(FA− and SU−) ≠ (FA+ and SU+)
Onset ED (years old)		21.68	9.95	20.84	9.80	19.89	9.96	0.556	
Duration ED (years)		7.98	8.34	11.94	10.00	15.05	9.74	0.001 *	All pairwise comparisons with *p* < 0.05
BMI (kg/m^2^)		25.34	10.59	28.05	9.42	28.09	9.22	0.160	
		*n*	%	*n*	%	*n*	%	*p*	Groups with significant differences
ED subtypes	AN	11	19.6%	17	10.7%	4	4.5%	0.001 *	All pairwise comparisons with *p* < 0.05
	BN	7	12.5%	71	44.7%	54	61.4%		
	BED	12	21.4%	38	23.9%	17	19.3%		
	OSFED	26	46.4%	33	20.8%	13	14.8%		

Note. AN: anorexia nervosa. BN: bulimia nervosa. BED: binge eating disorder. ED: eating disorder. OSFED: other specified feeding or eating disorder. FA−: food addiction negative screening score. FA+: food addiction positive screening score. SU−: lifetime substances use absent. SU+: lifetime substances use present. SD: standard deviation. * Bold: significant parameter.

**Table 2:** Distribution of the treatment outcomes.

	Total		FA− and SU−		FA+ or SU+		FA+ and SU+		FA+ or SU+ vs. FA− and SU−		FA+ and SU+ vs. FA− and SU−		FA+ and SU+ vs. FA+ or SU+	
	*n* = 303		*n* = 56		*n* = 159		*n* = 88							
	*n*	%	*n*	%	*n*	%	*n*	%	*p*	|*CV*|	*p*	|*CV*|	*p*	|*CV*|
Dropout	125	41.3%	21	37.5%	61	38.4%	43	48.9%	0.233	0.141	0.108	**0.205 ^†^**	0.421	0.107
Non-remission	24	7.9%	9	16.1%	11	6.9%	4	4.5%						
Partial remission	72	23.8%	12	21.4%	40	25.2%	20	22.7%						
Full remission	82	27.1%	14	25.0%	47	29.6%	21	23.9%						

Note. FA−: food addiction negative screening score. FA+: food addiction positive screening score. SU−: lifetime substances use absent. SU+: lifetime substances use present. ^†^ Bold: effect size into the range mild-moderate to large-high (|CV| > 0.15).

**Table 3:** Comparisons of the treatment outcomes in the study: results adjusted by the ED type.

	Total		FA− and SU−		FA+ or SU+		FA+ and SU+		FA+ or SU+ vs. FA− and SU−		FA+ & SU+ vs. FA− and SU−		FA+ and SU+ vs. FA+ or SU+	
	*n* = 303		*n* = 56		*n* = 159		*n* = 88							
	*n*	%	*n*	%	*n*	%	*n*	%	*p*	*OR*	*p*	*OR*	*p*	*OR*
Poor outcome	149	49.2%	30	53.6%	72	45.3%	47	53.4%	0.238	0.68	0.762	0.89	0.238	1.48
Good outcome	154	50.8%	26	46.4%	87	54.7%	41	46.6%		*1.47*		*1.12*		

Note. FA−: food addiction negative screening score. FA+: food addiction positive screening score. SU−: lifetime substances use absent. SU+: lifetime substances use present. Poor outcome: dropout or non-remission. Good outcome: partial remission or full remission. Italics font: inverse of the OR (1/OR).

**Table 4:** Predictors of poor treatment outcomes in the study.

*Subsample*	*Predictors*	*B*	*SE*	*p*	*OR*	*95%CI (OR)*		*H–L*	*NR^2^*
FA− and SU− (*n* = 56)	*TCI-R Persistence*	0.043	0.018	0.017	1.44	1.01	1.08	0.158	0.167
FA+ or SU+ (*n* = 159)	*Onset at the ED (years)*	−0.048	0.019	0.010	0.953	0.919	0.989	0.181	0.109
	*TCI-R harm avoidance*	0.021	0.009	0.018	1.021	1.004	1.039		
FA+ and SU+ (*n* = 88)	*Age (years)*	−0.049	0.021	0.021	0.952	0.914	0.993	0.338	0.084

Note. List of predictors: ED type, sociodemographic variables (gender, civil status, education, employment status, age), age at onset of ED, duration of ED, ED symptom severity level (EDI-2 total), psychopathology distress (SCL-90R GSI), impulsivity (UPPS-P total), and personality (TCI-R scales). H–L: Hosmer–Lemeshow test (*p*-value). *NR*^2^: Nagelkerke’s pseudo-R^2^.

## Data Availability

Data is not available in any repository. Contact with corresponding authors.

## Supplementary Materials

The following are available online at https://www.mdpi.com/article/10.3390/nu15132919/s1; Table S1: Descriptive statistics of the sample based on the ED types; Table S2: Distribution of the measures in the study; Table S3: Comparison between groups with a poor outcome; and Table S4: Comparison of the treatment outcomes in the study, stratified by the ED subtype.
