# Supplementary material for: The Role of Food Addiction and Lifetime Substance Use on Eating Disorder Treatment Outcomes
Source: Nutrients. 2023 Jun 27;15(13):2919. doi: 10.3390/nu15132919 (PMC10346594; doi:10.3390/nu15132919)
Supplement: Supplementary file 1 [file nutrients-15-02919-s001.zip › nutrients-2356577-supplementary.pdf]

nutrients

MDPI

Supplementary Materials

The Role of Food Addiction and Lifetime Substance Use on Eating Disorder Treatment Outcomes

Romina Miranda-Olivos, Zaida Agüera, Roser Granero, Susana Jiménez-Murcia, Montserrat Puig-Llobet, Maria Teresa Lluch-Canut, Ashley N. Gearhardt and Fernando Fernández-Aranda

Table S1. Descriptive of the sample based on the ED types.

		AN n = 32 *(10.6%)*		BN n = 132 *(43.6%)*		BED n = 67 *(22.1%)*		OSFED n = 72 *(23.7%)*		
		n	%	*n*	%	*n*	%	*n*	%	*p*
Gender	Women	30	93.8%	120	90.9%	59	88.1%	68	94.4%	0.558
	Men	2	6.3%	12	9.1%	8	11.9%	4	5.6%	
Marital status	Single	25	78.1%	86	65.2%	31	46.3%	59	81.9%	**0.001***
	Married - partner	5	15.6%	28	21.2%	23	34.3%	9	12.5%	
	Divorced - separated	2	6.3%	18	13.6%	13	19.4%	4	5.6%	
Education	Primary	15	46.9%	48	36.4%	26	38.8%	25	34.7%	0.538
	Secondary	14	43.8%	63	47.7%	26	38.8%	37	51.4%	
	University	3	9.4%	21	15.9%	15	22.4%	10	13.9%	
Employment	Unemployed	6	18.8%	23	17.4%	15	22.4%	16	22.2%	**0.021***
	Student	13	40.6%	31	23.5%	7	10.4%	22	30.6%	
	Employed	13	40.6%	78	59.1%	45	67.2%	34	47.2%	
		Mean	***SD***	***Mean***	***SD***	***Mean***	***SD***	***Mean***	***SD***	***p***
	Age (years-old)	28.19	11.42	31.39	10.98	40.79	12.81	28.24	10.65	**< 0.001***
	Onset ED (years-old)	17.47	5.74	19.59	8.94	26.03	12.28	19.28	8.70	**< 0.001***
	Duration ED (years)	10.53	10.04	12.57	9.45	15.18	11.47	9.11	8.13	**0.002***
	BMI (kg/m^2)	16.38	1.39	26.80	6.85	39.44	8.79	22.81	4.47	**< 0.001***
		n	%	*n*	%	*n*	%	*n*	%	*p*
FA	Negative	14	43.8%	8	6.1%	13	19.4%	27	37.5%	**< 0.001***
	Positive	18	56.3%	124	93.9%	54	80.6%	45	62.5%	
SU	Absent	25	78.1%	77	58.3%	49	73.1%	58	80.6%	**0.004***
	Present	7	21.9%	55	41.7%	18	26.9%	14	19.4%	
Group	FA– & SU–	11	34.4%	7	5.3%	12	17.9%	26	36.1%	**< 0 .001***
	FA+ or SU+	17	53.1%	71	53.8%	38	56.7%	33	45.8%	
	FA+ & SU+	4	12.5%	54	40.9%	17	25.4%	13	18.1%	

Note. AN: anorexia nervosa. BN: bulimia nervosa. BED: binge eating disorder. ED: eating disorder. OSFED: other specified feeding or eating disorder.

FA: current food addiction. FA–: food addiction negative screening score. FA+: food addiction positive screening score.

SU: lifetime substance use. SU–: lifetime substances use absent. SU+: lifetime substances use present.

Table S2. Distribution of the measures in the study.

Therapy outcome	FA– & SU–						FA+ or SU+						FA+ & SU+					
	Good		Poor				Good		Poor				Good		Poor			
	n = 26		*n* = 30				*n* = 87		*n* = 72				*n* = 41		*n* = 47			
	Mean	*SD*	*Mean*	*SD*	*p*	|*d*|	*Mean*	*SD*	*Mean*	*SD*	*p*	|*d*|	*Mean*	*SD*	*Mean*	*SD*	*p*	|*d*|
ED severity (EDI-2)																		
Drive thinness	12.19	5.93	9.83	6.73	0.173	0.37	15.07	4.80	15.22	4.93	0.843	0.03	14.71	5.43	16.43	4.65	0.114	0.34
Body dissatisfaction	16.69	6.92	11.87	9.13	**0.032***	**0.60†**	18.44	7.08	18.10	7.04	0.763	0.05	18.51	7.25	19.04	6.73	0.723	0.08
Interoc. awareness	9.50	6.74	8.33	8.29	0.570	0.15	12.70	6.61	12.86	6.82	0.881	0.02	14.44	6.66	13.51	7.18	0.533	0.13
Bulimia	3.42	4.22	2.93	4.13	0.663	0.12	8.90	5.17	8.78	5.00	0.884	0.02	9.44	4.58	9.57	4.65	0.891	0.03
Interper. distrust	5.46	5.27	5.97	4.99	0.714	0.10	5.61	4.85	6.58	4.89	0.211	0.20	6.15	5.43	6.70	4.52	0.601	0.11
Ineffectiveness	9.54	7.36	6.87	6.27	0.148	0.39	11.21	7.29	13.54	7.24	**0.045***	0.32	11.71	7.10	13.06	7.24	0.379	0.19
Maturity fears	7.92	4.80	6.43	4.52	0.237	0.32	7.69	5.22	9.11	5.59	0.100	0.26	8.51	5.96	9.26	6.29	0.573	0.12
Perfectionism	5.38	4.48	5.70	4.50	0.794	0.07	5.71	4.53	6.47	4.16	0.276	0.17	5.68	4.41	6.09	4.60	0.678	0.09
Impulse regulation	4.31	4.86	5.47	6.77	0.471	0.20	5.48	4.83	7.42	5.22	**0.017***	0.38	8.02	7.00	8.43	7.11	0.791	0.06
Ascetism	5.31	3.45	5.47	4.22	0.879	0.04	7.67	3.78	7.61	3.43	0.923	0.02	7.49	3.91	8.23	4.36	0.403	0.18
Social insecurity	7.00	5.61	5.83	5.48	0.435	0.21	7.33	5.08	8.74	4.60	0.072	0.29	7.95	4.71	8.94	5.23	0.358	0.20
Total score	86.73	39.98	74.70	47.60	0.315	0.27	105.80	36.48	114.43	34.43	0.130	0.24	112.61	38.73	119.26	39.43	0.429	0.17
Psychop. (SCL-90R)																		
Somatization	1.54	0.83	1.25	1.11	0.281	0.29	1.92	0.79	2.10	0.83	0.166	0.22	1.91	0.91	2.03	0.90	0.527	0.14
Obsessive-comp.	1.68	0.94	1.38	1.01	0.256	0.31	2.00	0.80	2.21	0.74	0.083	0.28	2.01	0.76	2.10	0.74	0.585	0.12
Interp. sensitivity	1.73	0.92	1.50	1.14	0.413	0.22	1.97	0.84	2.25	0.74	**0.026***	0.36	2.17	0.91	2.13	0.85	0.842	0.04
Depressive	1.88	0.87	1.56	1.06	0.232	0.33	2.28	0.81	2.54	0.74	**0.038***	0.33	2.44	0.90	2.51	0.77	0.724	0.08
Anxiety	1.20	0.73	1.19	1.01	0.978	0.01	1.73	0.79	1.91	0.76	0.148	0.23	1.76	0.92	1.99	0.90	0.246	0.25
Hostility	0.59	0.61	0.93	1.05	0.149	0.40	1.28	0.83	1.52	0.82	0.065	0.30	1.57	0.98	1.57	1.03	0.980	0.01
Phobic anxiety	0.74	0.84	0.68	0.88	0.796	0.07	1.00	0.86	1.20	0.91	0.143	0.23	1.14	0.94	1.32	1.03	0.388	0.19
Paranoia	1.06	0.70	1.16	0.87	0.648	0.12	1.46	0.81	1.56	0.79	0.412	0.13	1.56	0.81	1.50	0.94	0.761	0.07
Psychotic	1.10	0.79	0.77	0.72	0.108	0.44	1.33	0.72	1.58	0.69	**0.034***	0.34	1.46	0.77	1.53	0.75	0.693	0.08
GSI	1.40	0.68	1.22	0.87	0.402	0.23	1.78	0.62	1.99	0.57	**0.025***	0.36	1.88	0.73	1.97	0.72	0.542	0.13
PST	56.62	16.34	47.60	23.57	0.107	0.44	66.85	15.15	69.88	11.83	0.169	0.22	66.20	15.91	69.15	13.98	0.356	0.20
PSDI	2.11	0.51	2.09	0.67	0.921	0.03	2.33	0.47	2.53	0.48	**0.009***	0.42	2.46	0.56	2.49	0.53	0.774	0.06
Impulsivity (UPPS-P)																		
Lack premeditation	22.15	5.37	20.90	4.59	0.350	0.25	23.57	6.32	23.30	6.14	0.780	0.04	25.05	6.45	25.43	6.46	0.785	0.06
Lack perseverance	22.19	5.22	18.83	4.68	**0.014***	**0.68†**	23.36	5.53	24.06	4.83	0.404	0.13	25.56	5.84	24.96	6.91	0.662	0.09
Sensation seeking	24.96	8.32	27.97	8.45	0.187	0.36	25.43	8.64	25.51	7.95	0.951	0.01	26.41	7.94	28.72	9.47	0.223	0.26
Positive urgency	24.81	8.05	25.93	9.70	0.642	0.13	28.61	8.97	28.83	8.38	0.874	0.03	30.17	9.77	33.13	11.20	0.194	0.28

Negative urgency	29.42	7.01	27.70	7.44	0.379	0.24	33.99	6.71	35.87	5.29	0.056	0.31	36.76	6.48	35.66	6.29	0.423	0.17
Total	123.54	21.98	121.33	23.30	0.718	0.10	134.94	21.75	137.55	21.43	0.452	0.12	143.95	19.86	147.87	28.03	0.457	0.16
Personality (TCI-R)																		
Novelty seeking	92.28	12.13	97.53	14.04	0.148	0.40	99.02	17.87	99.27	15.13	0.927	0.01	104.78	19.37	107.02	15.64	0.550	0.13
Harm avoidance	113.52	17.94	103.67	21.01	0.070	**0.50†**	116.59	21.17	124.24	16.93	**0.015***	0.40	119.76	18.78	122.51	18.57	0.492	0.15
Reward dependence	99.20	18.63	101.27	18.52	0.683	0.11	101.11	15.98	98.20	14.25	0.233	0.19	100.59	18.46	98.53	16.58	0.584	0.12
Persistence	103.72	19.95	117.50	17.72	**0.009***	**0.73†**	109.64	21.66	105.61	19.09	0.221	0.20	103.90	21.87	105.32	19.83	0.751	0.07
Self-directedness	126.84	17.78	131.50	17.79	0.338	0.26	115.61	18.79	111.37	19.61	0.168	0.22	112.07	18.08	107.26	19.09	0.229	0.26
Cooperativeness	133.76	16.57	136.83	17.25	0.506	0.18	136.57	16.77	132.86	16.02	0.159	0.23	130.59	16.52	131.98	16.89	0.697	0.08
Self-transcendence	65.28	22.46	64.87	17.28	0.939	0.02	67.85	16.90	64.46	13.28	0.171	0.22	66.68	13.68	66.53	14.70	0.961	0.01

Note. FA–: food addiction negative screening score. FA+: food addiction positive screening score.

SU–: substances use absent. SU+: substances use present.

* Bold: significant parameter. † Bold: effect size into the range mild-moderate to large-high.

Table S3. Comparison between the groups with poor outcome.

	FA− & SU−		FA+ or SU+		FA+ & SU+		FA− & SU− vs FA+ or SU+		FA− & SU− vs FA+ & SU+		FA+ or SU+ vs FA+ & SU+	
	n = 30		*n = 72*		*n = 47*							
	Mean	*SD*	*Mean*	*SD*	*Mean*	*SD*	*p*	*|d|*	*p*	*|d|*	*p*	*|d|*
ED severity (EDI-2)												
Drive for thinness	9.83	6.73	15.22	4.93	16.43	4.65	**0.001***	**0.91†**	**0.001***	**1.14†**	0.224	0.25
Body dissatisfaction	11.87	9.13	18.10	7.04	19.04	6.73	**0.001***	**0.76†**	**0.001***	**0.89†**	0.498	0.14
Interoceptive awareness	8.33	8.29	12.86	6.82	13.51	7.18	**0.005***	**0.60†**	**0.003***	**0.67†**	0.633	0.09
Bulimia	2.93	4.13	8.78	5.00	9.57	4.65	**0.001***	**1.28†**	**0.001***	**1.51†**	0.370	0.17
Interpersonal distrust	5.97	4.99	6.58	4.89	6.70	4.52	0.555	0.12	0.513	0.15	0.895	0.03
Ineffectiveness	6.87	6.27	13.54	7.24	13.06	7.24	**0.001***	**0.99†**	**0.001***	**0.91†**	0.719	0.07
Maturity fears	6.43	4.52	9.11	5.59	9.26	6.29	**0.030***	**0.53†**	**0.034***	**0.52†**	0.892	0.02
Perfectionism	5.70	4.50	6.47	4.16	6.09	4.60	0.418	0.18	0.707	0.08	0.637	0.09
Impulse regulation	5.47	6.77	7.42	5.22	8.43	7.11	0.149	0.32	**0.043***	0.43	0.386	0.16
Ascetism	5.47	4.22	7.61	3.43	8.23	4.36	**0.013***	**0.56†**	**0.003***	**0.64†**	0.396	0.16
Social insecurity	5.83	5.48	8.74	4.60	8.94	5.23	**0.008***	**0.57†**	**0.009***	**0.58†**	0.831	0.04
Total score	74.70	47.60	114.43	34.43	119.26	39.43	**0.001***	**0.96†**	**0.001***	**1.02†**	0.510	0.13
Psychopathology (SCL-90R)												
Somatization	1.25	1.11	2.10	0.83	2.03	0.90	**0.001***	**0.87†**	**0.001***	**0.77†**	0.682	0.08
Obsessive-compulsive	1.38	1.01	2.21	0.74	2.10	0.74	**0.001***	**0.94†**	**0.001***	**0.81†**	0.464	0.15
Interpersonal sensitivity	1.50	1.14	2.25	0.74	2.13	0.85	**0.001***	**0.79†**	**0.002**	**0.62†**	0.436	0.16
Depressive	1.56	1.06	2.54	0.74	2.51	0.77	**0.001***	**1.07†**	**0.001***	**1.02†**	0.843	0.04
Anxiety	1.19	1.01	1.91	0.76	1.99	0.90	**0.001***	**0.80†**	**0.001***	**0.83†**	0.624	0.10
Hostility	0.93	1.05	1.52	0.82	1.57	1.03	**0.005***	**0.62†**	**0.004***	**0.61†**	0.761	0.06
Phobic anxiety	0.68	0.88	1.20	0.91	1.32	1.03	**0.011***	**0.59†**	**0.004***	**0.68†**	0.506	0.12
Paranoia	1.16	0.87	1.56	0.79	1.50	0.94	**0.030***	**0.49†**	0.083	0.38	0.713	0.07
Psychoticism	0.77	0.72	1.58	0.69	1.53	0.75	**0.001***	**1.15†**	**0.001***	**1.03†**	0.725	0.07
GSI	1.22	0.87	1.99	0.57	1.97	0.72	**0.001***	**1.05†**	**0.001***	**0.94†**	0.867	0.03
PST	47.60	23.57	69.88	11.83	69.15	13.98	**0.001***	**1.19†**	**0.001***	**1.11†**	0.803	0.06
PSDI	2.09	0.67	2.53	0.48	2.49	0.53	**0.001***	**0.77†**	**0.002***	**0.66†**	0.660	0.09
Impulsivity (UPPS-P)												
Lack premeditation	20.90	4.59	23.30	6.14	25.43	6.46	0.067	0.44	**0.001***	**0.81†**	0.060	0.34
Lack perseverance	18.83	4.68	24.06	4.83	24.96	6.91	**0.001***	**1.10†**	**0.001***	**1.04†**	0.389	0.15
Sensation seeking	27.97	8.45	25.51	7.95	28.72	9.47	0.189	0.30	0.706	0.08	**0.048***	0.37
Positive urgency	25.93	9.70	28.83	8.38	33.13	11.20	0.169	0.32	**0.002**	**0.69†**	**0.019***	0.43
Negative urgency	27.70	7.44	35.87	5.29	35.66	6.29	**0.001***	**1.27†**	**0.001***	**1.16†**	0.852	0.04
Total UPPS-P	121.33	23.30	137.55	21.43	147.87	28.03	**0.002***	**0.72†**	**0.001***	**1.03†**	**0.024***	0.41
Personality (TCI-R)												
Novelty seeking	97.53	14.04	99.27	15.13	107.02	15.64	0.598	0.12	**0.008***	**0.64†**	**0.007***	**0.50†**
Harm avoidance	103.67	21.01	124.24	16.93	122.51	18.57	**0.001***	**1.08†**	**0.001***	**0.95†**	0.617	0.10
Reward dependence	101.27	18.52	98.20	14.25	98.53	16.58	0.378	0.19	0.464	0.16	0.911	0.02
Persistence	117.50	17.72	105.61	19.09	105.32	19.83	**0.005***	**0.65†**	**0.007***	**0.65†**	0.936	0.01
Self-directedness	131.50	17.79	111.37	19.61	107.26	19.09	**0.001***	**1.08†**	**0.001***	**1.31†**	0.254	0.21
Cooperativeness	136.83	17.25	132.86	16.02	131.98	16.89	0.272	0.24	0.211	0.28	0.778	0.05
Self-transcendence	64.87	17.28	64.46	13.28	66.53	14.70	0.900	0.03	0.626	0.10	0.453	0.15

Note. FA– & SU–: food addiction negative screening and substances use absent.

FA+ | SU+: food addiction positive screening or substances use present.

FA+ & SU+: food addiction positive screening and substances use present.

* Bold: significant parameter. † Bold: effect size into the range mild-moderate to large-high.

Table S4. Comparison of the treatment outcomes in the study stratified by the ED subtype.

		**FA− & SU−**		**FA+ or SU+**		**FA+ & SU+**			
		n	**%**	***n***	**%**	***n***	**%**	***p***	**|*CV*|**
AN	Poor outcome	7	63.6%	8	47.1%	3	75.0%	0.497	**0.209**†
	Good outcome	4	36.4%	9	52.9%	1	25.0%		
BN	Poor outcome	3	42.9%	38	53.5%	32	59.3%	0.647	0.081
	Good outcome	4	57.1%	33	46.5%	22	40.7%		
BED	Poor outcome	5	41.7%	12	31.6%	5	29.4%	0.763	0.090
	Good outcome	7	58.3%	26	68.4%	12	70.6%		
OSFED	Poor outcome	15	57.7%	14	42.4%	7	53.8%	0.363	0.142
	Good outcome	11	42.3%	19	57.6%	6	46.2%		

Note. AN: anorexia nervosa. BN: bulimia nervosa. BED: binge eating disorder. OSFED: other specified feeding or eating disorder.

FA−: food addiction negative screening score. FA+: food addiction positive screening score.

SU−: substances use absent. SU+: substances use present.

Poor outcome: dropout or non-remission. Good outcome: partial remission or full remission.

* Bold: significant parameter. † Bold: effect size into the range mild-moderate to large-high (|*CV*| > 0.15).
